# The association between nutritional assessment tools and CT-assessed sarcopenia in abdominal aortic aneurysm: a cross-sectional study

**DOI:** 10.3389/fnut.2025.1679038

**Published:** 2025-10-29

**Authors:** Jiashu Yao, Yepeng Zhang, Wenqing Chen, Xiangrui Li, Guangyan Wu, Jie Wang, Xiaotian Chen, Bo Gao, Min Zhou

**Affiliations:** ^1^Department of Clinical Nutrition, Nanjing Drum Tower Hospital, The Affiliated Hospital of Nanjing University Medical School, Nanjing, Jiangsu, China; ^2^Department of Vascular Surgery, Nanjing Drum Tower Hospital, The Affiliated Hospital of Nanjing University Medical School, Nanjing, China

**Keywords:** nutritional assessment, sarcopenia, abdominal aortic aneurysm, nutrition, undernutrition

## Abstract

**Objective:**

To evaluate the correlation between computed tomography (CT)-assessed sarcopenia and nutritional assessment tools in patients with abdominal aortic aneurysm (AAA).

**Methods:**

In this single-center retrospective study, 232 AAA patients admitted to our hospital between January 2022 and December 2024 were included. Patients’ demographic characteristic were collected. Nutritional assessment tools were calculated, including the nutritional risk screening 2002 (NRS2002), controlling nutritional status (CONUT) score, geriatric nutritional risk index (GNRI), and prognostic nutritional index (PNI). Sarcopenia was diagnosed through CT measured skeletal muscle mass index at the third lumbar vertebra level, including rectus abdominis, internal/external obliques, transversus abdominis, erector spinae, quadratus lumborum, and psoas muscles. Logistic regression analysis was used to assess the association between nutritional assessment tools and sarcopenia in AAA patients. The optimal cutoff values of each nutritional assessment tools for screening sarcopenia in AAA patients were determined based on receiver operating characteristic (ROC) curve analysis.

**Results:**

Multivariate regression analysis revealed that NRS2002 (odds ratio [OR] 1.83, 95% confidence interval [CI] 1.27 ~ 2.63), CONUT (OR 1.40, 95% CI 1.18 ~ 1.66), and GNRI (OR 0.90, 95% CI 0.85 ~ 0.95) were independently associated with sarcopenia in AAA patients (*p* < 0.05). NRS2002 (AUC = 0.735) outperformed CONUT (AUC = 0.613) and PNI (AUC = 0.600) in sarcopenia screening, showing comparable accuracy to GNRI (AUC = 0.694), with superior specificity (92.11% vs. 57.89%) but lower sensitivity (48.45% vs. 78.76%) than GNRI.

**Conclusion:**

NRS2002 and GNRI demonstrated potential as supportive assessment methods. Their clinical utility as independent screening tools in sarcopenia among AAA patients remained limited due to their respective low sensitivity/specificity.

## 1 Introduction

Sarcopenia, a syndrome characterized by progressive deterioration of skeletal muscle mass, strength, and physical performance, demonstrates distinct higher prevalence rates in elderly hospitalized populations. Clinical evidence reveals a male predominance in prevalence rates (1). This skeletal muscle disorder constitutes a major public health concern due to its substantial impact on functional capacity and health-related quality of life. Severe sarcopenia may lead to serious consequences such as functional impairment, physical disability, or even death (1). Multiple studies have reported the correlations between sarcopenia and prognosis in patients with abdominal aortic aneurysms (AAA). Sarcopenia has been associated with significantly increased mortality risk (2–4), and higher postoperative complication rates after AAA repair (5). Additionally, patients undergoing endovascular repair are more likely to develop sarcopenia, with an incidence rate up to 67% (6). Therefore, sarcopenia screening in AAA patients allows early detection of high-risk groups, providing a scientific basis for prompt initiation of nutrition and exercise programs aimed at reversing sarcopenia.

Consensus guidelines recommend that the diagnosis of sarcopenia should incorporate at least one of three core parameters: lean body mass, muscle strength, and physical performance (7, 8). Computed Tomography (CT) is the gold-standard technique for quantifying muscle mass in sarcopenia, with key metrics including skeletal muscle area (SMA), skeletal muscle index (SMI), and muscle radiation attenuation (MRA) (8, 9). Despite providing reliable skeletal muscle mass data, the use of CT imaging in routine clinical practice is limited due to its high cost and radiation exposure risks. Additionally, the CT-based assessment of sarcopenia requires the identification and extraction of specific CT across-sectional slices for the delineation and measurement of muscle tissues. This process is time-consuming and laborious, therefore, it has certain limitations when rapid screening for sarcopenia is required. Dual-energy X-ray absorptiometry (DXA) and bioelectrical impedance analysis (BIA) serve as alternative methods for estimating muscle mass. However, DXA faces challenges with equipment costs and weaker correlations (10), while BIA measurements are confounded by hydration status, skin temperature, and body composition variations (1). In contrast, nutritional assessment tools are calculated based on data such as height, weight, hematological indices, and questionnaires related to diet and weight changes. These parameters are typically collected upon admission, with the process of calculation being relatively convenient and rapid. Hence, integrating these nutritional scoring indicators for verification is a worthwhile consideration. If the verification yield positive results, they can be deployed for swiftly screening and guiding nutritional status assessments during follow-up. These indicators come with merits like convenience, economic viability, and freedom from radiation. At this juncture, CT is inappropriate for routine follow-up screening.

Malnutrition is an established risk factor for sarcopenia (1, 11). This metabolic imbalance exhibits complex multifactorial mechanisms associated to sarcopenia (12). Standardized nutritional assessment tools should therefore be employed for screening, coupled with interventions combining personalized exercise training and nutrition plans to mitigate sarcopenia risk (13, 14). Notably, there is limited evidence evaluating the association between nutritional assessment tools and sarcopenia risk in AAA patients.

The nutrition risk screening 2002 (NRS2002) demonstrates high sensitivity and specificity for malnutrition identification (15) and correlates with prognosis in patients with acute myocardial infarction (16). Nutritional assessment tools, including the prognostic nutritional index (PNI), the controlling nutritional status (CONUT), and the geriatric nutritional risk index (GNRI), have been validated as predictive and prognostic factors for cardiovascular diseases and AAA (17–19). These scores are readily calculated using routine hematological and anthropometric data. Previous studies have shown that the GNRI demonstrates superior diagnostic accuracy for sarcopenia in patients with type 2 diabetes and individuals undergoing cardiac surgery (20, 21), while the NRS 2002 is associated with the occurrence of sarcopenia in cancer patients (22). However, no studies have explored the association between these nutritional indices and sarcopenia in AAA patients. This study investigates the relationship between CT-diagnosed sarcopenia and nutritional assessment tools in AAA patients.

## 2 Methods

### 2.1 Study design and participants

**Figure 1:**
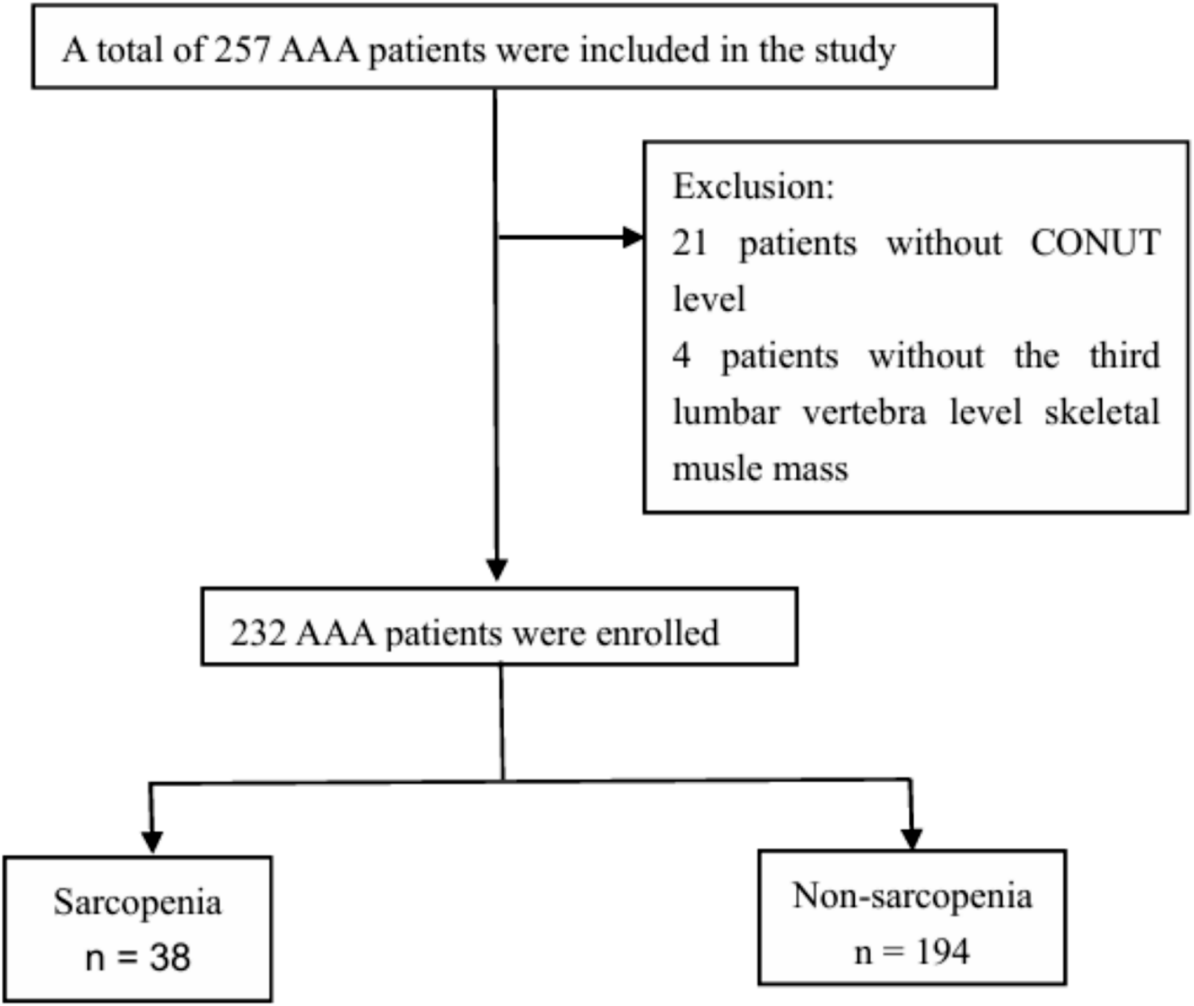
Flow chart of participant selection.

This single-center retrospective study included patients for AAA at our hospital from January 2022 to December 2024. Among 257 initially screened AAA cases, 25 patients (21 with incomplete CONUT scores and 4 lacking third lumbar vertebra [L3]-level skeletal muscle mass measurements) were excluded per predefined criteria. Two hundred thirty-two patients were ultimately included (Figure 1). This study complied with the ethical principles of the Declaration of Helsinki and was approved by the Ethics Committee of Nanjing Drum Tower Hospital, Affiliated Hospital of Nanjing University Medical School (authorization/protocol 2022–086-02). Written informed consent was obtained from all enrolled patients prior to data collection. The Ethics Committee of Nanjing Drum Tower Hospital Affiliated to Nanjing University approved the waiver of informed consent for patients who were unable to provide informed consent themselves or whose family members were unable to provide informed consent.

### 2.2 Data collection and follow-up

Demographic information including age, height, weight, smoking history, and comorbidities was documented. Hematological parameters (routine blood tests, coagulation function, hepatic and renal function) within 24 h of admission were collected.

All participants underwent abdominal CT scans within 24 h of admission, with 4 cases excluded due to poor imaging quality of abdominal musculature at L3 level. The poor quality was caused by factors such as the compression or traction of the aneurysm. Radiological evaluation included documentation of abdominal aortic aneurysm diameter and analysis of axial images obtained at the mid-L3 vertebral level. The single-slice scan at the L3 level has been established as the optimal compromise site for assessing whole-body skeletal muscle, visceral adipose tissue, and subcutaneous adipose tissue (23–25). It serves as the gold standard for the quantitative assessment of trunk muscles in research (26) and is recommended for the diagnostic evaluation of sarcopenia (27). DICOM files were processed using Slice-O-Matic 5.0 (TomoVision, Canada) for skeletal muscle area (SMA) segmentation, including rectus abdominis, internal/external obliques, transversus abdominis, erector spinae, quadratus lumborum, and psoas muscles. The software can delineate specific tissues by utilizing HU thresholds. The CT HU thresholds were set as follows: −29 to +150 for skeletal muscle area, −190 to −30 for subcutaneous adipose tissue, and −150 to −50 for visceral adipose tissue. The software automatically calculates the skeletal muscle cross-sectional area (SMA, cm^2^) and skeletal muscle density (SMD, Hounsfield unit [HU]). The skeletal muscle index (SMI) was calculated as SMA (cm^2^) divided by the square of height (m^2^). Similarly, the cross-sectional areas of adipose tissue were normalized by the square of height (cm^2^/m^2^), and these values were termed the subcutaneous adipose tissue (SAT) index and visceral adipose tissue (VAT) index (28, 29). The cross-sectional area (cm^2^) was automatically calculated, and the tissue boundaries were manually adjusted. All CT images were analyzed by two trained observers.

### 2.3 Assessment of sarcopenia

We adopted the diagnostic criteria from previous studies in Chinese populations to define sarcopenia. The specific diagnostic threshold criteria were: SMI < 39 cm^2^/m^2^ (male) and <31.1 cm^2^/m^2^ (female) for BMI < 25 kg^2^/m^2^; adjusted thresholds of <46.2 cm^2^/m^2^ (male) and <34.2 cm^2^/m^2^ (female) for BMI ≥ 25 kg^2^/m^2^ (24).

### 2.4 Nutritional assessment tools

#### 2.4.1 NRS2002

The NRS2002 scale assessed nutritional risk by evaluating disease severity (mild, moderate and severe), impairment of nutritional status (mild, moderate and severe), and age (Supplementary Table 1). NRS2002 ≥ 3 identified nutritional risk or malnutrition (30).

#### 2.4.2 CONUT

The CONUT score was calculated from serum albumin concentration, cholesterol level, and lymphocyte count (31) (Supplementary Table 1). Briefly, each parameter is scored as follows: albumin concentration: ≥3.5 mg/dL: 0 points; 3.0–3.49 mg/dL: 2 points; 2.5–2.99 mg/dL: 4 points; and <2.5 mg/dL: 6 points. Total lymphocyte count: ≥1,600/mm^3^: 0 points; 1,200-1599/mm^3^: 1 point; 800-1199/mm^3^: 2 points; and <800/mm^3^: 3 points. Total cholesterol level scoring: ≥180 mg/dL: 0 points; 140–179 mg/dL: 1 point; 100–139 mg/dL: 2 points; <100 mg/dL: 3 points. The sum of these scores was defined as the CONUT score.

#### 2.4.3 GNRI

The GNRI was calculated by the formula: GNRI = 14.89 × serum albumin level (g/dL) + 41.7 × (current weight [kg]/ideal weight [kg]) (32). The formula for calculating ideal weight was: ideal weight = 22 * square of height (m).

#### 2.4.4 PNI

The prognostic nutritional index (PNI) was determined based on admission laboratory parameters. The PNI calculation follows this standardized formula: PNI = 10 × serum albumin level (g/dl) + 0.005 × total lymphocyte count (per mm^3^) (33).

### 2.5 Statistical analysis

Normally distributed continuous variables were expressed as mean ± SD, while skewed data were reported as median (interquartile range). Categorical variables were described as counts (percentage). Intergroup comparisons were performed using independent samples t-test, Mann–Whitney U test, and analysis of variance. Covariates in multivariate analysis were selected based on variables with *p*-value < 0.05 in univariate analysis and those previously demonstrated to be strongly associated with sarcopenia (1, 34).

The screening performance of NRS2002, CONUT, GNRI, and PNI scores were assessed using receiver operating characteristic (ROC) curves. ROC curves and area under the curve (AUC) were compared using DeLong’s method (35). The optimal cutoff values were derived from ROC curve analysis using Youden index calculation with maximum likelihood estimation (36). Statistical significance was defined as two-tailed *p*-values < 0.05. The Hosmer–Lemeshow goodness-of-fit test was employed to assess model calibration. Non-significant results (*p* > 0.05) were interpreted as evidence of adequate fit between predicted probabilities and observed outcomes. All analyses were conducted with SPSS 25.0 (SPSS Inc., Chicago, IL, United States) and MedCalc 15.2.2 statistical software.

## 3 Results

### 3.1 Characteristics of study population

The baseline demographic and clinical characteristics of the patients were summarized in Table 1. The mean age of the patients was 71.1 ± 10.4 years, with males accounting for 80.2%. The prevalence of sarcopenia was 16.38%, among which 86.8% were male. The sarcopenia group demonstrated higher age, NRS2002, CONUT, HDL-C, and D-dimer levels compared to the non-sarcopenia group (*p* < 0.05). However, the sarcopenia group exhibited lower GNRI, serum albumin, SMA, SMD, SMI, SATA, and SAT indices compared to the non-sarcopenia group (*p* < 0.05). Remaining baseline characteristics showed no significant intergroup differences.

**Table 1:** Basic characteristics of the study population.

Variables	Overall (*n* = 232)	Non-sarcopenia (*n* = 194)	Sarcopenia (*n* = 38)	*P*
Age, years	71.1 ± 10.4	70.3 ± 10.3	75.7 ± 10.0	**0.003**
Gender (male/female)	186/46 (80.2/19.8)	153/41 (78.9/21.1)	33/5 (86.8/13.2)	0.259
Body weight, kg	66.0 ± 11.2	66.4 ± 10.1	64.5 ± 15.6	0.473
Height, cm	168.3 ± 7.7	168.0 ± 7.6	169.5 ± 7.9	0.276
BMI, kg/m^2^	23.3 ± 3.2	23.4 ± 2.9	22.3 ± 4.4	0.125
Current or ex-smoking, *n*(%)	97 (41.8)	83 (42.8)	14 (36.8)	0.497
T2DM, *n*(%)	53 (22.8)	43 (22.2)	10 (26.3)	0.577
AAA diameter, cm	6.0 ± 6.7	5.6 ± 2.0	8.5 ± 16.0	0.272
SMA, cm^2^	132.7 ± 27.6	137.8 ± 26.2	107.0 ± 18.8	**<0.001**
SMD, HU	33.6 ± 7.9	34.4 ± 7.8	29.5 ± 6.9	**<0.001**
SMI, cm^2^/m^2^	46.7 ± 8.6	48.7 ± 7.7	36.4 ± 4.3	**<0.001**
VATA, cm^2^	123.0 ± 65.4	125.2 ± 65.5	111.7 ± 64.7	0.246
VAT index, cm^2^/m^2^	43.4 ± 23.0	44.4 ± 23.1	38.7 ± 22.1	0.169
SATA, cm^2^	134.0 ± 58.7	138.1 ± 59.6	112.9 ± 49.6	**0.015**
SAT index, cm^2^/m^2^	47.8 ± 22.4	49.4 ± 22.8	39.4 ± 17.9	**0.011**
NRS2002, points	3.0(2.0, 4.0)	3.0 (1.0, 4.0)	4.0 (3.0, 4.0)	**<0.001**
NRS2002 ≥ 2, *n*(%)	135 (58.2)	100 (51.6)	35 (92.1)	**<0.001**
COUNT, points	3.39 ± 2.64	3.21 ± 2.55	4.32 ± 2.95	**0.028**
CONUT ≥ 5, *n*(%)	208 (89.7)	172 (88.7)	36 (94.7)	0.405
GNRI, points	97.9 ± 10.5	99.1 ± 10.2	92.0 ± 10.3	**<0.001**
GNRI ≤ 92, *n*(%)	68 (29.3)	46 (23.7)	22 (57.9)	**<0.001**
PNI, points	47.8 ± 24.0	48.3 ± 25.1	45.0 ± 17.3	0.435
PNI ≤ 38, *n*(%)	129 (55.6)	103 (53.1)	26 (68.4)	0.082
Serum albumin, g/L	36.6 ± 5.4	37.1 ± 5.3	33.9 ± 5.3	**<0.001**
TC, mg/dL	152.4(123.9, 176.1)	149.8 (123.4, 174.5)	157.4 (126.8, 183.7)	0.216
TG, mg/dL	105.4 (79.7, 152.3)	106.7 (80.6, 154.1)	96.5 (72.6, 121.3)	0.171
HDL-C, mg/dL	36.4 (29.8, 43.7)	35.6 (29.0, 43.0)	39.9 (34.4, 48.0)	**0.045**
LDL-C, mg/dL	89.6 (63.6, 110.7)	87.9 (62.3, 109.9)	93.7 (73.1, 120.0)	0.128
ALT, U/L	13.9(10.3, 20.2)	14.1 (10.4, 22.9)	13.05 (9.1, 16.9)	0.071
AST, U/L	17.7 (14.4, 22.6)	17.9 (14.7, 23.0)	16.5 (13.0, 20.0)	0.113
Scr, mg/dL	0.9 (0.7, 1.2)	0.9 (0.7, 1.2)	0.9 (0.7, 1.1)	0.615
eGFR, ml/min/1.73m^2^	88.8 (59.9, 109.8)	88.8 (59.8, 109.7)	87.0 (65.6, 109.7)	0.878
FPG, mg/dL	89.9 (79.1, 111.6)	88.6 (80.4, 112.3)	101.2 (76.7, 110.7)	0.788
CRP, mg/dL	6.8 (3.3, 32.2)	6.3 (3.3, 27.1)	6.9 (3.5, 35.1)	0.171
D-Dimer, mg/L	3.3 (1.3, 9.0)	3.0 (1.3, 7.9)	5.9 (2.7, 17.3)	**0.005**
Length of stay, d	14.5 ± 10.0	14.9 ± 10.4	12.8 ± 7.9	0.237

Data are expressed as median [25, 75%], Mean ± SD, or number (%).

BMI, body mass index; T2DM, type 2 diabetes mellitus (T2DM); SMA, skeletal muscle mass area; SMD, skeletal muscle mass density; SMI: skeletal muscle index; VATA, visceral adipose tissue area; VAT, visceral adipose tissue; SATA, subcutaneous adipose tissue area; SAT, subcutaneous adipose tissue; NRS2002, Nutritional risk screening 2002; CONUT, controlling nutritional status; GNRI, geriatric nutritional risk index; PNI, prognostic nutritional index; FPG, fasting plasma glucose; TC, total cholesterol; TG, triglyceride; HDL-C, high density lipoprotein-cholesterol; LDL-C, low-density lipoprotein-cholesterol; ALT, alanine aminotransferase; AST, aspartate aminotransferase; Scr, serum creatinine concentration; CRP, C-reactive protein; eGFR, estimated glomerular filtration rate.The bold entries are statistical significance. *P* ≤ 0.05 are statistical significance.

### 3.2 Univariate and multivariate logistic regression analysis of the association between nutritional assessment tools and sarcopenia in abdominal aortic aneurysm patients

The univariate logistic regression analysis (Table 2) identified three screening tools significantly associated with sarcopenia in AAA patients: NRS2002, CONUT, and GNRI. According to the previously described method, variables with *p* < 0.05 in the univariate analysis and those previously demonstrated to be closely associated with sarcopenia were included in different models. Subsequent multivariate analysis further validated these associations through two distinct modeling approaches. In Model 1 (adjusted for age, sex and BMI), all three screening tools maintained screening significance for sarcopenia (*p* < 0.05). Model 2 (incorporating BMI, LDL-C, ALT, smoking history and eGFR based on model 1) demonstrated persistent correlations, with NRS2002 (OR = 1.83, 95% CI 1.27 ~ 2.63, *p* = 0.001), CONUT (OR = 1.40, 95%CI 1.18 ~ 1.66, *p* < 0.001), and GNRI (OR = 0.90, 95%CI 0.85 ~ 0.95, *p* < 0.001). Notably, PNI failed to show statistical significance in either models (Table 2).

**Table 2:** Univariate and multivariate logistic regression analysis on associations of the nutritional assessment tools with sarcopenia in abdominal aortic aneurysm participants.

Variables	Unadjusted OR (95% CI)	*P*	Model 1 OR (95% CI)	*P*	Model 2 OR (95% CI)	*P*
NRS2002	1.94 (1.43 ~ 2.64)	**<0.001**	1.75 (1.24 ~ 2.48)	**0.002**	1.83 (1.27 ~ 2.63)	**0.001**
CONUT	1.16 (1.02 ~ 1.31)	**0.021**	1.17 (1.02 ~ 1.33)	**0.024**	1.40 (1.18 ~ 1.66)	**<0.001**
GNRI	0.93 (0.90 ~ 0.97)	**<0.001**	0.93 (0.88 ~ 0.97)	**0.003**	0.90 (0.85 ~ 0.95)	**<0.001**
PNI	0.99 (0.97 ~ 1.01)	0.445	0.99 (0.97 ~ 1.02)	0.589	0.98 (0.93 ~ 1.02)	0.243

NRS2002, Nutritional risk screening 2002; CONUT, controlling nutritional status; GNRI, geriatric nutritional risk index; PNI, prognostic nutritional index; BMI, body mass index; eGFR, estimated glomerular filtration rate; CI: confidence interval; OR: odds ratio OR, odds ratio; CI, confidential intervals.

Model 1: Age, sex and BMI.

Model 2: age, sex, BMI, LDL-C, ALT, smoking history and eGFR.The bold entries are statistical significance. *P* ≤ 0.05 are statistical significance.

The NRS2002 (*r* = 0.31, *p* < 0.001) and CONUT (*r* = 0.15, *p* = 0.028) showed significant positive correlations with the prevalence of sarcopenia, while the GNRI (*r* = −0.25, *p* < 0.001) demonstrated a significant negative correlation with sarcopenia prevalence. The correlations remained consistent even after adjusting for potential confounding variables, including age, sex, and BMI, which were reported to be closely related to the prevalence of sarcopenia (1, 37) (Supplementary Table 2).

### 3.3 Comparison of the value of nutritional assessment tools in screening sarcopenia in abdominal aortic aneurysm patients

**Figure 2:**
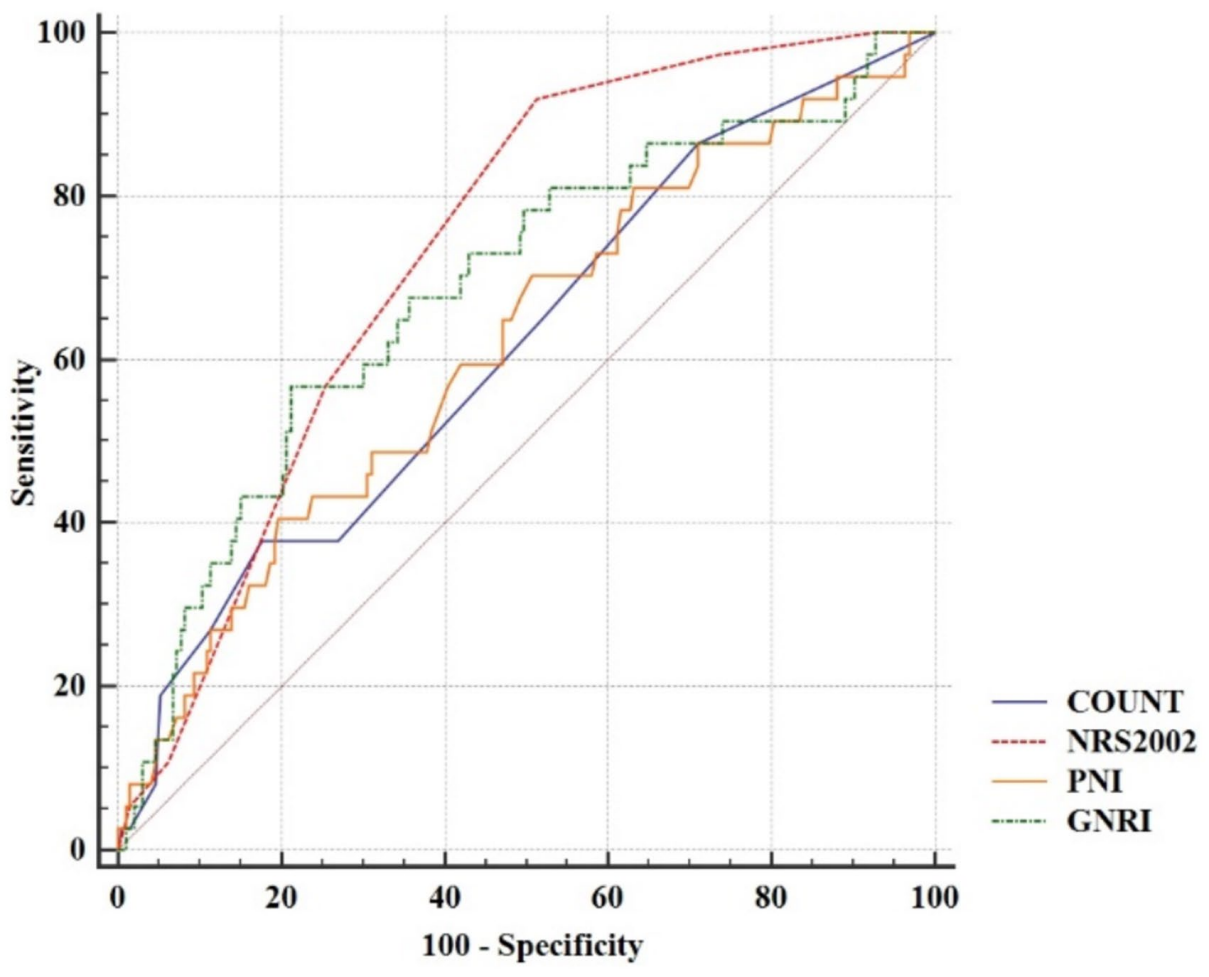
Receiver-operating characteristic (ROC) curves of the nutritional assessment tools for screening sarcopenia. The area under the receiver operating characteristic curve (AUC) values were calculated to assess the screening performance of four nutritional assessment tools for sarcopenia: nutritional risk screening 2002 (NRS2002, red dashed line), controlling nutritional status (CONUT, blue solid line), geriatric nutritional risk index (GNRI, green dashed line), and prognostic nutritional index (PNI, orange solid line). Table 3 presented the AUC statistical significance comparisons and detailed the screening thresholds alongside their corresponding sensitivity and specificity parameters.

The AUCs and optimal cutoff values of NRS2002, CONUT, GNRI, and PNI for screening sarcopenia in AAA patients were showed in Figure 2 and Table 3. Among all participants, the AUCs ranked in descending order as follows: NRS2002 > GNRI > CONUT > PNI. Tested by DeLong’s method, the AUC value of NRS2002 was significantly higher than those of CONUT and PNI (*p* < 0.05), but showed no significant difference compared with GNRI. The ROC curve of NRS2002 revealed an optimal cutoff value of 2 (AUC = 0.735, 95% CI 0.64 ~ 0.79), with sensitivity and specificity of 48.45 and 92.11%, respectively. For GNRI, the optimal cutoff value was 91.4 (AUC = 0.694, 95% CI 0.63 ~ 0.75), demonstrating sensitivity and specificity of 78.76 and 57.89%, respectively. All Hosmer-Lemeshow statistical tests yielded *p*-values > 0.05, indicating good calibration.

**Table 3:** Screening performance of the nutritional assessment tools for screening sarcopenia.

Variables	AUC (95% CI)	Cut-off	Specificity (%)	Sensitivity (%)	*P*	HLS	HLS *P-*value
NRS2002	0.735 (0.64 ~ 0.79)	>2	92.11	48.45	**<0.001**	6.13	0.633
CONUT	0.613 (0.55 ~ 0.68)^#^	≥5	37.84	81.96	**0.026**	5.77	0.673
GNRI	0.694 (0.63 ~ 0.75)	≤91.4	57.89	78.76	**<0.001**	4.88	0.771
PNI	0.600 (0.53 ~ 0.66)^#^	≤38.3	39.47	79.90	0.058	14.60	0.067

NRS2002, Nutritional risk screening 2002; CONUT, controlling nutritional status; GNRI, geriatric nutritional risk index; PNI, prognostic nutritional index; HLS, Hosmer–Lemeshow goodness-of-fit test.

# respectively compared with NRS2002, *p* < 0.05.The bold entries are statistical significance. *P* ≤ 0.05 are statistical significance.

## 4 Discussion

Sarcopenia is characterized by a widespread and progressive loss of skeletal muscle mass and strength, associated with metabolic, physiological, and functional impairments as well as adverse clinical outcomes following various types of surgery (1). Sarcopenia has proved to be associated with the survival rate and postoperative complications in AAA patients (6, 38, 39). The meta-analysis conducted by Nana et al. (40) demonstrated that compared to non-sarcopenic patients, sarcopenic patients exhibited significantly increased risks of midterm mortality (25% [95% CI 0.19 ~ 0.31] vs. 13% [95% CI 0.03 ~ 0.29], OR 1.11, 95% CI 0.21 ~ 2.44; *p* < 0.001, I^2^ = 88.32%) and postoperative spinal cord ischemia (19% [95% CI 4 ~ 34] vs. 7% [95% CI 5 ~ 20], OR 1.80, 95% CI 0.17 ~ 3.78; *I*^2^ = 82.4%). Furthermore, sarcopenia was also associated with increased long-term mortality and postoperative acute kidney injury incidence (5, 41).

Muscle mass can be assessed through various methods including BIA, magnetic resonance imaging (MRI), CT, DXA, and ultrasound. However, MRI, CT, and DXA are complex and costly, while BIA is susceptible to confounding factors such as skin temperature (1, 8, 42). Additionally, although nearly all AAA patients underwent preoperative abdominal CT scans, CT-based sarcopenia assessment requires identification and extraction of specific CT slices to delineate and measure muscle tissues. This process is time-consuming and labor-intensive. Therefore, CT-based sarcopenia assessment has certain limitations when rapid sarcopenia screening is required. In contrast, nutritional assessment tools are calculated based on parameters routinely collected during hospitalization, including height, weight, hematological indicators, and questionnaires regarding diet and weight changes. The calculation process for these readily available parameters is relatively convenient and efficient.

Emerging evidence substantiates associations between nutritional assessment tools and sarcopenia across diverse clinical settings (20–22, 43–47). Wang et al.’s (22) cohort study of 1,637 colorectal cancer patients identified NRS 2002 as a dual indicator, revealing significant associations with both reduced SMI (OR 2.56, 95% CI 1.56 ~ 4.22) and SMD (OR 5.43, 95% CI 3.43 ~ 8.60). GNRI has also been validated to correlate with sarcopenia risk in dialysis recipients, cirrhotic patients, oncology patients, type 2 diabetics, and geriatric inpatients (20, 43–46). Güç et al.’s (46) investigation of 185 colorectal cancer patients established CONUT as an independent prognostic marker for sarcopenia (HR 2.01, 95% CI 1.06 ~ 3.73). Concurrently, a Turkish multicenter study demonstrated s PNI and GNRI significantly depressed among sarcopenic elderly hospitalized patients (≥65 years) compared to non-sarcopenic patients (48).

Current studies have predominantly focused on the prognostic significance of nutritional assessment tools or sarcopenia in patients with AAA (49, 50). To date, no studies have systematically elucidated the correlation between nutritional assessment tools and sarcopenia in patients with AAA. This cross-sectional study evaluated the association between clinically common nutritional assessment tools (NRS2002, CONUT, GNRI, and PNI) and sarcopenia. Through ROC curve analysis, we determined optimal screening cut-off values for each tool and explored their performance in this specific population, providing insights for clinical application.

Aligning with established evidence, we confirmed the association of NRS2002, CONUT, and GNRI for sarcopenia in AAA patients. However, PNI demonstrated no independent association for sarcopenia in our study through multivariate regression analysis.

Malnutrition plays a pivotal role in the pathogenesis of sarcopenia (14). The NRS 2002, an established instrument for nutritional risk assessment, demonstrates predictive capacity for malnutrition development and clinical outcomes (12, 51). While existing evidence demonstrates a significant association between NRS 2002 and sarcopenia among colorectal cancer patients (22), its clinical applicability in AAA patients remains underinvestigated. A Chinese colorectal cancer cohort study established diagnostic cutoffs for low SMI as ≤36.2 cm^2^/m^2^ for males and ≤29.6 cm^2^/m^2^ for females, reporting an AUC value of 0.711 (95% CI 0.682 ~ 0.739) for NRS 2002 in predicting sarcopenia (22). Our study extends these findings to AAA patients, demonstrating comparable screening accuracy (AUC = 0.735, 95%CI 0.64 ~ 0.83) while maintaining significant specificity.

The GNRI is composed of a combination of serum albumin concentration and body weight, both closely associated with sarcopenia (11, 52). Previous studies have demonstrated an L-shaped negative correlation between GNRI and sarcopenia in middle-aged and elderly populations, with an optimal inflection point at GNRI = 91.935 (53). Based on ROC curve analysis, we determined the optimal GNRI cutoff value for screening sarcopenia in AAA patients as 91.4, which aligns closely with the above finding. Studies in cardiac surgery patients demonstrated that the GNRI achieved an AUC of 0.716 (95%CI 0.664 ~ 0.768) in diagnosing sarcopenia, with specificity and sensitivity of 65.1 and 67.7%, respectively (21). Our study revealed that the GNRI exhibited an AUC value of 0.694 (95% CI 0.63 ~ 0.75) for identifying sarcopenia in AAA patients, which was consistent with the aforementioned research. The sensitivity and specificity of GNRI screening for sarcopenia in AAA patients in our study were 57.89 and 78.76%, respectively.

Among the four nutritional assessment tools evaluated, CONUT and PNI integrate immune-nutritional markers including albumin and lymphocytes, but demonstrate limitations in analysis of body composition and nutritional intake. These compositional differences may constrain their screening efficacy. Previous studies have demonstrated significant correlations between body weight and sarcopenia prevalence (11, 37). While serum albumin reflects nutritional status, its levels are influenced by three factors: reduced intake, impaired synthesis, and increased protein loss. Notably, inadequate dietary intake is one of the primary cause of chronic malnutrition in elderly populations (54). As a key intervention for sarcopenia, high-protein intake shows positive correlations with muscular strength and physical activity in sarcopenic patients (1, 55), and may decrease the mortality risk associated with hypoalbuminemia in elderly sarcopenia populations (56). Our findings revealed substantial screening superiority of NRS2002 over CONUT and PNI in AAA patients (AUC: 0.735 vs. 0.631 and 0.600 respectively). This may be attributed to the fact that NRS2002 incorporates key sarcopenia screening indicators such as age, weight variation, and dietary status (57, 58), suggesting that integrating multi-dimensional parameters could enhance the performance of sarcopenia screening.

The NRS2002 and CONUT were cumulative scores derived from categorical classifications with defined maxima (7 and 12 points respectively). In contrast, the GNRI and PNI were calculated from formula transformations of continuous variables and did not have upper bounds. This difference in scoring methodology may impact sensitivity and specificity. For instance, the NRS2002 and CONUT have fewer inflection points in the ROC curve. This phenomenon has also been observed in the studies of Wang et al. (22) and Pan et al. (59). In future research, perhaps combining NRS2002 and GNRI or incorporating different parameters to construct a new model could be explored to improve the efficiency of screening sarcopenia in AAA patients.

This study has several limitations. Firstly, this study is a single-center retrospective survey with a relatively small sample size. Therefore, these findings necessitate further validation through the expansion of the sample size for various subgroup analyses. Secondly, the diagnosis of sarcopenia was based on L3-SMI cut-off values adjusted for gender and BMI in Chinese populations previously reported (24), without through methods such as handgrip strength or gait speed assessment. Thirdly, we did not analyze the medication history, particularly the use of statins, which may cause bias in research results. Fourthly, although both NRS2002 and GNRI demonstrate relatively high AUC values for screening sarcopenia in AAA patients, the lower sensitivity of NRS2002 and the lower specificity of GNRI might limit their practical value in clinical screening. It would be worthwhile to further explore the potential application of composite indicators combining these two tools in sarcopenia screening among AAA patients. Fifthly, the size and location of AAA may affect muscle area at the L3 level through the compression or traction of the aneurysm. Our study has excluded patients with poor muscle quality due to the abovementioned reasons. Therefore, the findings of this study do not applicable to this specific patient population. Finally, the follow-up duration was constrained. Future research should establish a universal definition of sarcopenia in AAA patients and elucidate the relationship between sarcopenia and nutritional status through well-designed large-scale studies.

## 5 Conclusion

In this study, we evaluated the association between nutritional assessment tools and sarcopenia in AAA patients. NRS 2002, GNRI, and CONUT were significantly associated with sarcopenia in AAA patients. Both NRS2002 and GNRI demonstrated some potential as supportive assessment methods, but their clinical utility as stand-alone screening tools in sarcopenia of AAA patients remained limited due to their respective low sensitivity/specificity.

## Data Availability

The original contributions presented in the study are included in the article/Supplementary material, further inquiries can be directed to the corresponding authors.
