# Supplementary material for: The association between nutritional assessment tools and CT-assessed sarcopenia in abdominal aortic aneurysm: a cross-sectional study
Source: Front Nutr. 2025 Oct 29;12:1679038. doi: 10.3389/fnut.2025.1679038 (PMC12605111; doi:10.3389/fnut.2025.1679038)
Supplement: Supplementary file 1 [file Table_1.docx]

**Supplement Table 1.** Nutritional risk screening 2002 scale.

| **Severity of disease** | |
| --- | --- |
| Score 0 | Normal nutritional requirements |
| Score 1 (Mild) | Hip fracture; chronic patients; in particular with acute complications: cirrhosis, COPD; chronic hemodialysis; diabetes; oncology |
| Score 2 (Moderate) | Major abdominal surgery; stroke; severe pneumonia; hematologic malignancy |
| Score 3 (Severe) | Head injury; bone marrow transplantation; intensive care patients |
| **Impaired nutritional status** | |
| Score 0 | Normal nutritional status |
| Score 1 (Mild) | Weight loss > 5% in 3 months or food intake below 50-75% of normal requirement in preceding week |
| Score 2 (Moderate) | Weight loss > 5% in 2 months or BMI 18.5-20.5 + impaired general condition or food intake below 25%-50% of normal requirement in preceding week |
| Score 3 (Severe) | Weight loss > 5% in 1 month (≈ > 15% in 3 months) or BMI < 18.5% + impaired general condition or food intake 0-25% of normal requirement in preceding week |
| **Age** | |
| Score 0 | Age ≤ 70 yeas |
| Score 1 | Age > 70 years |
| **Total Score = severity of disease + impaired nutritional status + age** | |
| < 3 | No nutritional intervention but need weekly evaluation |
| ≥ 3 | Start nutritional support |

Abbreviations: COPD = chronic obstructive pulmonary disease; BMI = body mass index.

**Supplement Table 2.** Controlling nutritional status (CONUT) scores

|  | Score | | | |
| --- | --- | --- | --- | --- |
| Parameter | Normal status | Low risk | Moderate risk | Severe risk |
| Serum albumin, g/dL | ≥3.5 | 3.0-3.49 | 2.50-2.99 | <2.5 |
| Albumin score | 0 | 2 | 4 | 6 |
| Total cholesterol, mg/dL | ≥180 | 140-179 | 100-139 | <100 |
| Cholesterol score | 0 | 1 | 2 | 3 |
| Lymphocytes, count/mL | ≥1600 | 1200-1599 | 800-1199 | <800 |
| Lymphocyte score | 0 | 1 | 2 | 3 |
| Screening total score | 0-1 | 2-4 | 5-8 | 9-12 |

**Supplement Table 3.** Correlations between nutritional assessment tools with sarcopenia.

|  | Sarcopenia |
| --- | --- |
| Simple correlations | |
| NRS2002 | 0.31 (< 0.001) |
| CONUT | 0.15 (0.028) |
| GNRI | - 0.25 (< 0.001) |
| PNI | - 0.13 (0.051) |
| Age, sex and body mass index-adjusted partial correlations | |
| NRS2002 | 0.21 (0.001) |
| CONUT | 0.15 (0.022) |
| GNRI | - 0.19 (0.005) |
| PNI | - 0.04 (0.581) |

Data are presented as r (*P*).
